# Supplementary material for: Construction of an immunogenic cell death-based risk score prognosis model in breast cancer
Source: Front Genet. 2022 Dec 13;13:1069921. doi: 10.3389/fgene.2022.1069921 (PMC9792780; doi:10.3389/fgene.2022.1069921)
Supplement: Supplementary file 1 [file DataSheet1.docx]

**Supplementary Materials**

Table S1

| Gene | Protein | Function |
| --- | --- | --- |
| *CALR* | Calreticulin | DAMPs |
| *HMGB1* | High mobility group protein B1 | DAMPs |
| *ANXA1* | Annexin A1 | DAMPs |
| *PDIA3* | Protein disulfide-isomerase A3 | DAMPs |
| *HSPA4* | Heat shock 70 kDa protein 4 | DAMPs |
| *HSP90AA1* | Heat shock protein HSP 90-alpha | DAMPs |
| *IFNG* | Interferon gamma | DAMPs |
| *CXCL10* | C-X-C motif chemokine 10 | DAMPs |
| *PANX1* | Pannexin-1 | DAMPs |
| *ROCK1* | Rho-associated protein kinase 1 | DAMPs |
| *LRP1* | Prolow-density lipoprotein receptor-related protein 1 | DAMPs Receptor |
| *TLR2* | Toll-like receptor 2 | DAMPs Receptor |
| *TLR3* | Toll-like receptor 3 | DAMPs Receptor |
| *TLR4* | Toll-like receptor 4 | DAMPs Receptor |
| *TLR7* | Toll-like receptor 7 | DAMPs Receptor |
| *TLR8* | Toll-like receptor 8 | DAMPs Receptor |
| *TLR9* | Toll-like receptor 9 | DAMPs Receptor |
| *AGER* | Advanced glycosylation end product-specific receptor | DAMPs Receptor |
| *FPR1* | fMet-Leu-Phe receptor | DAMPs Receptor |
| *P2RY2* | P2Y purinoceptor 2 | DAMPs Receptor |
| *P2RX7* | P2X purinoceptor 7 | DAMPs Receptor |
| *CXCR3* | C-X-C chemokine receptor type 3 | DAMPs Receptor |
| *ZBP1* | Z-DNA-binding protein 1 | DAMPs Receptor |
| *IFNAR1* | Type I interferon receptor | DAMPs Receptor |
| *CASP8* | Caspase-8 | danger signaling component |
| *BAX* | Apoptosis regulator BAX | danger signaling component |
| *BAK* | Bcl-2 homologous antagonist/killer | danger signaling component |
| *YKT6* | Synaptobrevin homolog YKT6 | danger signaling component |
| *EIF2AK3* | Eukaryotic translation initiation factor 2-alpha kinase 3 | danger signaling component |
| *BCAP31* | B-cell receptor-associated protein 31 | danger signaling component |
| *PIK3CA* | Phosphatidylinositol 4,5-bisphosphate 3-kinase catalytic subunit alpha isoform | danger signaling component |
| *ATG5* | Autophagy protein 5 | danger signaling component |
| *ATG7* | Ubiquitin-like modifier-activating enzyme ATG7 | danger signaling component |
| *LAMP1* | Lysosome-associated membrane glycoprotein 1 | danger signaling component |
| *BECN1* | Beclin-1 | danger signaling component |
| *IL10* | Interleukin-10 | ICD associated immune effector |
| *IL6* | Interleukin-6 | ICD associated immune effector |
| *TNF* | Tumor necrosis factor | ICD associated immune effector |
| *IFIH1* | Interferon-induced helicase C domain-containing protein 1 | ICD associated immune effector |
| *DDX58* | Antiviral innate immune response receptor RIG-I | ICD associated immune effector |
| *AIM2* | Interferon-inducible protein AIM2 | ICD associated immune effector |
| *IL1R1* | Interleukin-1 receptor type 1 | Purinergic Receptor-Inflammasome-interleukin1β axis |
| *IL1B* | Interleukin-1 beta | Purinergic Receptor-Inflammasome-interleukin1β axis |
| *NLRP3* | NACHT, LRR and PYD domains-containing protein 3 | Purinergic Receptor-Inflammasome-interleukin1β axis |
| *ENTPD1* | CD39 | Subversion of danger signaling |
| *CCL2* | C-C motif chemokine 2 | T cell effector |
| *CXCL1* | Growth-regulated alpha protein | T cell effector |
| *IL17A* | Interleukin-17A | T cell effector |
| *IL17RA* | Interleukin-17 receptor A | T cell effector |
| *PRF1* | Perforin-1 | T cell effector |
| *CCR2* | C-C chemokine receptor type 2 | T cell effector |
| *CXCR2* | C-X-C chemokine receptor type 2 | T cell effector |
| *CLEC9A* | C-type lectin domain family 9 member A | APCs effector |
| *LY96* | Lymphocyte antigen 96 | Toll-like Receptor Signaling |
| *TFAM* | Transcription factor A, mitochondrial | Transcription factor |

Table S1 Immunogenic cell death associated genes(1-3)

Abbreviations: ICD, Immunogenic cell death; APC, antigen presenting cells; DAMPs, damage-associated molecular patterns

References

1. Galluzzi L, Buqué A, Kepp O, Zitvogel L, Kroemer G. Immunogenic cell death in cancer and infectious disease. Nat Rev Immunol. 2017;17(2).

2. Galluzzi L, Vitale I, Warren S, Adjemian S, Agostinis P, Martinez AB, et al. Consensus guidelines for the definition, detection and interpretation of immunogenic cell death. J Immunother Cancer. 2020;8(1).

3. Garg AD, De Ruysscher D, Agostinis P. Immunological metagene signatures derived from immunogenic cancer cell death associate with improved survival of patients with lung, breast or ovarian malignancies: A large-scale meta-analysis. Oncoimmunology. 2016;5(2):e1069938.

Table S2

|  | baseMean | log2FoldChange | lfcSE | stat | pvalue | padj |
| --- | --- | --- | --- | --- | --- | --- |
| AIM2 | 43.323 | 2.171 | 0.180 | 12.034 | 2.35E-33 | 1.32E-32 |
| ANXA1 | 4106.611 | -2.305 | 0.107 | -21.575 | 3.1E-103 | 1.2E-101 |
| BAX | 673.922 | 1.203 | 0.070 | 17.188 | 3.27E-66 | 5E-65 |
| CALR | 19794.650 | 1.047 | 0.063 | 16.495 | 4E-61 | 5.38E-60 |
| CCL2 | 584.101 | -1.100 | 0.125 | -8.774 | 1.72E-18 | 5.37E-18 |
| CLEC9A | 8.262 | -0.599 | 0.156 | -3.834 | 0.000126 | 0.000193 |
| CXCR2 | 13.471 | -1.965 | 0.124 | -15.837 | 1.74E-56 | 2.03E-55 |
| CXCR3 | 93.274 | 1.657 | 0.152 | 10.876 | 1.5E-27 | 6.75E-27 |
| DDX58 | 1361.699 | 0.902 | 0.097 | 9.299 | 1.42E-20 | 4.84E-20 |
| IL1B | 99.628 | -0.616 | 0.132 | -4.665 | 3.09E-06 | 5.24E-06 |
| IL1R1 | 1790.282 | -1.026 | 0.106 | -9.694 | 3.19E-22 | 1.16E-21 |
| LRP1 | 9676.920 | -1.703 | 0.096 | -17.793 | 7.97E-71 | 1.36E-69 |
| P2RY2 | 195.610 | 0.839 | 0.123 | 6.809 | 9.85E-12 | 2.28E-11 |
| PIK3CA | 623.484 | -0.666 | 0.077 | -8.659 | 4.77E-18 | 1.46E-17 |
| TLR3 | 187.811 | -1.107 | 0.106 | -10.469 | 1.19E-25 | 5E-25 |
| TLR4 | 488.386 | -1.537 | 0.090 | -17.149 | 6.42E-66 | 9.76E-65 |
| YKT6 | 2357.923 | 0.684 | 0.049 | 14.063 | 6.37E-45 | 5.22E-44 |
| ZBP1 | 72.690 | 1.925 | 0.169 | 11.376 | 5.49E-30 | 2.72E-29 |

Table S2 Differential analysis results for 18 ICD-related DEGs

Figure S1


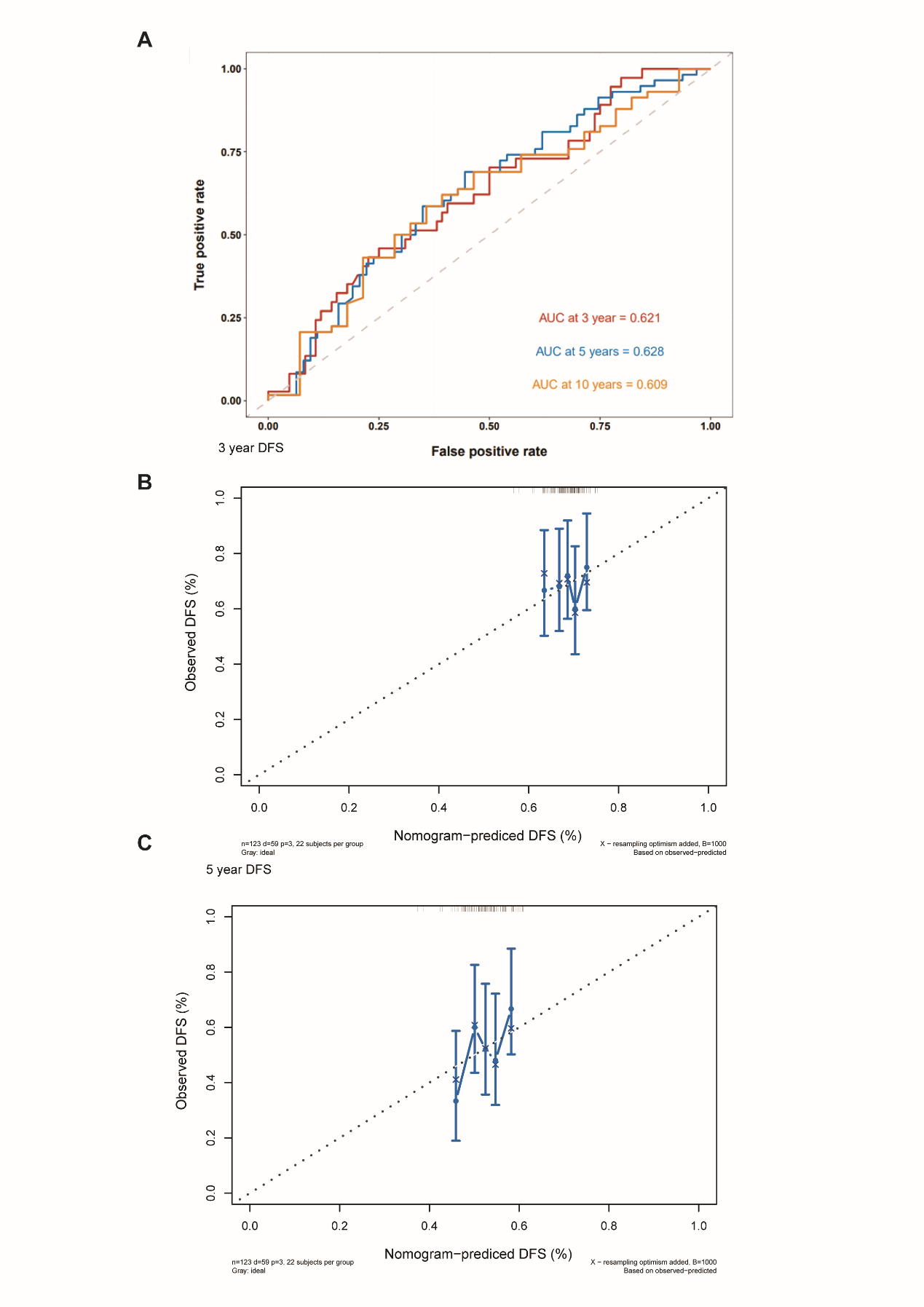
 Figure S1 External validation of ICD-based prognostic model

(A) ROC curves of the ICD-based prognostic model in the external validation cohort. (B) Calibration curves of the ICD-based prognostic model. The calibration curve for 10-year DFS is not provided due to the lack of subjects that have enough observation time in the validation cohort.

Figure S2


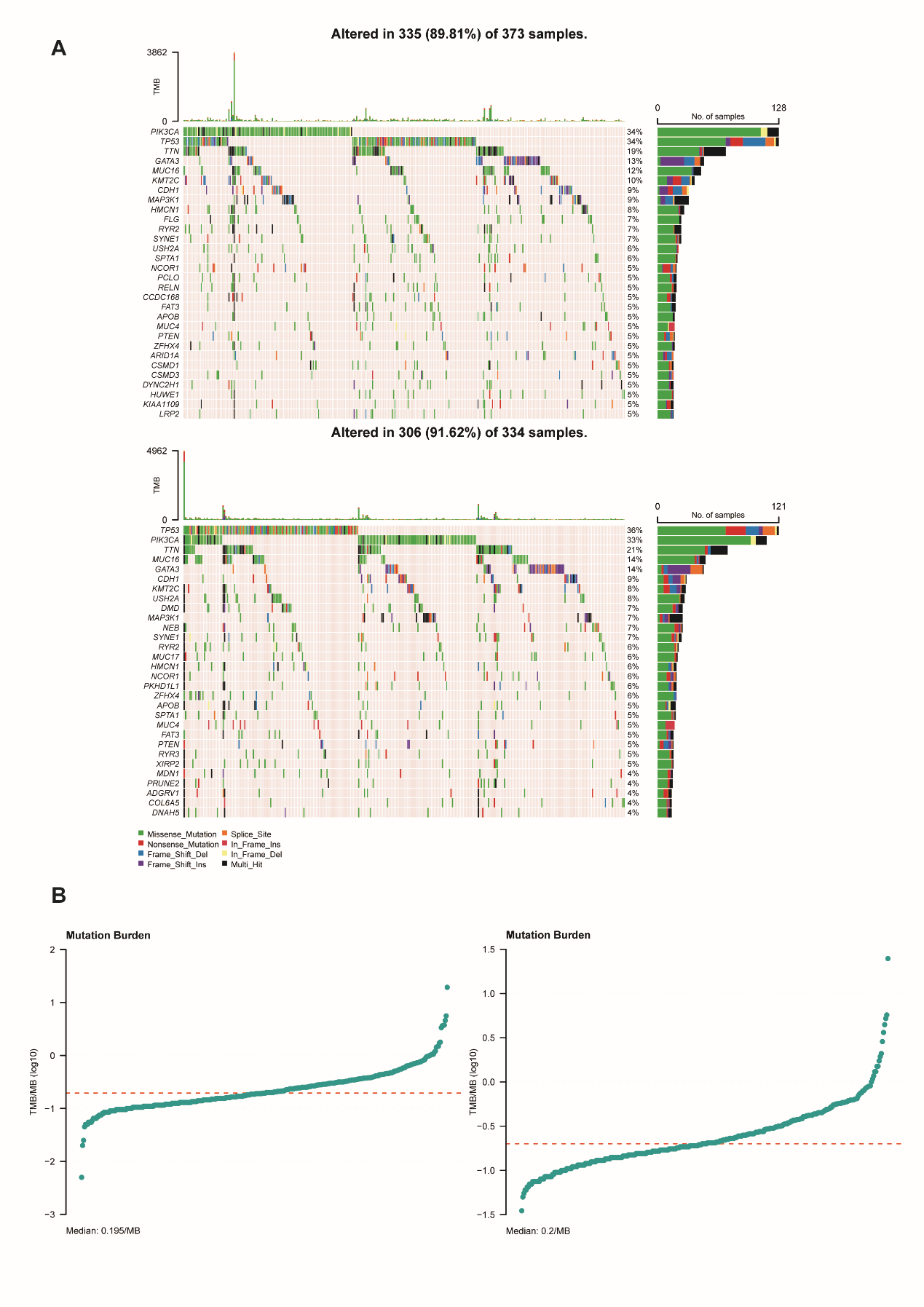


Figure S2 Tumor mutation burden of two risk groups

(A) Mutation landscapes of high-risk group (top) and low-risk group (bottom). (B) Tumor mutation burden of high-risk (left) and low-risk group (right).

Figure S3


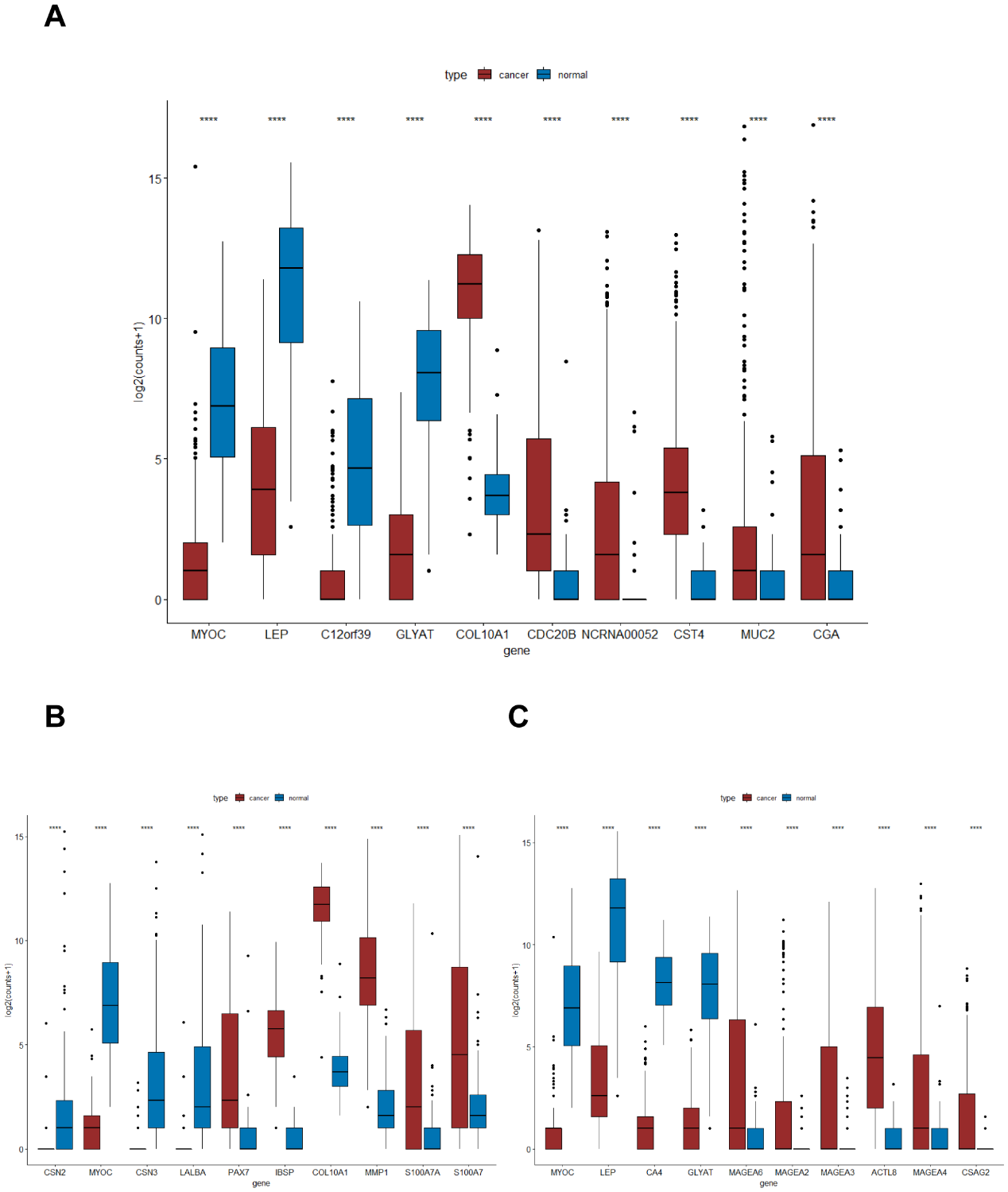


Figure S3 Differential analysis in breast cancer molecular subgroups

(A) Boxplot of the top 5 most up-regulated and down- regulated genes in HR+HER2- breast cancer. (B) Boxplot of the top 5 most up-regulated and down- regulated genes in HER2+ breast cancer.

(C) Boxplot of the top 5 most up-regulated and down- regulated genes in triple negative breast cancer. ***P < 0.001
